# Efficacy of sacituzumab govitecan versus treatment of physician’s choice in previously treated HR+ and HER2− mBC: a meta-analysis of TROPiCS-02 and EVER-132-002 trials

**DOI:** 10.1177/17588359251320285

**Published:** 2025-03-14

**Authors:** Oleg Gluz, Binghe Xu, Rita Nanda, Anandaroop Dasgupta, Ankita Kaushik, Wendy Verret, Akanksha Sharma, Barinder Singh, Hope S. Rugo

**Affiliations:** Breast Center Niederrhein, Evangelical Hospital Johanniter Bethesda, Moenchengladbach, Germany; Department of Medical Oncology, Cancer Hospital, Chinese Academy of Medical Sciences, Beijing, China; Section of Hematology/Oncology, Department of Medicine, The University of Chicago Comprehensive Cancer Center, Chicago, IL, USA; Gilead Sciences, Inc., Foster City, CA, USA; Gilead Sciences, Inc., Foster City, CA, USA; Gilead Sciences, Inc., Foster City, CA, USA; Pharmacoevidence, SAS Nagar Mohali, Punjab, India; Pharmacoevidence, London, UK; Helen Diller Family Comprehensive Cancer Center, University of California, 1825 4th Street, San Francisco, CA 94158, USA

**Keywords:** efficacy, EVER-132-002, HR+/HER2− mBC, metastatic breast cancer, sacituzumab govitecan, TROPiCS-02

## Abstract

**Background and objective:**

TROPiCS-02 and EVER-132-002 are phase III randomized controlled trials (RCTs) comparing sacituzumab govitecan (SG) to treatment of physician’s choice (TPC) in patients with hormone receptor-positive and human epidermal growth factor receptor 2 negative (HR+/HER2−) locally recurrent inoperable or metastatic breast cancer (mBC) who have progressed after two to four prior chemotherapy regimens. TROPiCS-02 enrolled mainly non-Asian patients, whereas EVER-132-002 consisted of only Asian participants. In this study, we compared the efficacy outcomes for SG to TPC via a meta-analysis of the two trials.

**Methods:**

Preferred Reporting Items for Systematic Reviews and Meta-Analyses of Individual Participant Data (PRISMA-IPD) guidelines were followed. IPD from the trials were assessed for integrity, consistency, imbalances, or missing values and were combined to estimate pooled and relative treatment effects for comparison of overall survival (OS), progression-free survival (PFS), duration of response (DOR), objective response rate (ORR), and clinical benefit rate (CBR) in the overall, cyclin-dependent kinase 4/6 inhibitor (CDK4/6i) pre-treated, and fast-progressors population (defined as the subgroup of patients with duration of prior CDK4/6i ⩽12 months).

**Results:**

In general, TROPiCS-02 and EVER-132-002 had a similar distribution of baseline population characteristics except for prior CDK4/6i treatment and geography. In the overall meta-analytic model, SG showed a significant improvement over TPC in PFS (hazard ratio (HR), 0.62 (95% confidence interval (CI): 0.50–0.77); *p* < 0.001) and OS (HR, 0.66 (95% CI: 0.55–0.80); *p* < 0.001). Similar patterns in efficacy were observed in both fast-progressors as well as patients previously treated with CDK4/6i. In the overall population, SG was associated with statistically significantly higher ORR (rate ratio (RR), 1.45 (95% CI: 1.09–1.95); *p* = 0.012), CBR (RR, 1.59 (95% CI: 1.28–1.97); *p* < 0.001), and DOR (HR, 0.55 (95% CI: 0.32–0.95); *p* = 0.032) compared to TPC.

**Conclusion:**

In conclusion, this meta-analysis confirms that SG significantly improves clinical outcomes in patients with HR+/HER2− mBC, including those pre-treated with CDK4/6i and fast-progressors when compared to TPC. These findings extend previous research, supporting the integration of SG into clinical practice guidelines at a global level for treating the HR+/HER2− mBC population irrespective of status and duration of prior CDK4/6i exposure.

## Background and objective

Breast cancer is considered to be the most common malignant tumor and the leading cause of death in females worldwide.^1,2^ Between 2017 and 2021, the hormone receptor-positive and human epidermal growth factor receptor 2 negative (HR+/HER2−; HER2− defined as IHC0, IHC1 positive, or IHC2 positive and ISH negative) subtype of breast cancer emerged as the most prevalent, with an age-adjusted rate of 90.0 new cases per 100,000 women.^3^ Approximately, 75% of breast cancer patients are identified with HR+/HER2− breast cancer subtype.^4^ In the United States, the estimated number of new breast cancer cases in 2024 is 313,510, while the estimated death numbers are 42,780.^5^ Moreover, 45.4% of the 2.3 million breast cancers diagnosed globally in 2020 were from Asia. The age-standardized incidence rate per 100,000 females was lowest for Asia (36.8), and highest for Northern America (89.4).^6^ For HR+/HER2− breast cancer patients, endocrine therapy stands as a cornerstone treatment approach for systemic treatment, followed by therapeutic agents such as aromatase inhibitors, selective estrogen receptor degraders, and selective estrogen receptor modulators which have also shown evidence of yielding clinical benefits.^4^ Furthermore, in patients with refractory or rapidly progressing cancer or extensive visceral metastatic disease, chemotherapy emerges as a first-line treatment option. When dealing with endocrine-resistant/refractory disease, treatment options include single-agent chemotherapy such as anthracyclines, taxanes, antimetabolites, and microtubule inhibitors.^7–9^ However, the efficacy of chemotherapy in treating HR+/HER2− metastatic breast cancer (mBC) is limited. There persists a significant gap in addressing the needs of patients with HR+/HER2− mBC who have undergone previous endocrine therapy and chemotherapy for locally advanced or metastatic disease.

Sacituzumab govitecan (SG) is a first-in-class antibody–drug conjugate (ADC), consisting of a humanized monoclonal antibody, hRS7 immunoglobulin G (IgG)1κ, which is linked to SN-38, a topoisomerase I inhibitor, via a specific hydrolyzable CL2A linker. This antibody component targets the human trophoblast cell-surface antigen 2 (Trop-2), enabling SN-38 to internalize and activate its anti-tumor actions.^10–12^ SG is distinguished by its high specificity for Trop-2 with a drug-to-antibody ratio of 7.6:1. SG has been granted approval in multiple countries for treating adult patients with unresectable locally advanced or metastatic triple-negative breast cancer (mTNBC) who have received two or more prior systemic therapies, at least one of them for metastatic disease and also in patients with unresectable locally advanced or HR+/HER2− mBC who have received at least two prior systemic treatments in the metastatic setting.^13^

Two randomized, open-label, and multicentered phase III clinical trials, namely TROPiCS-02 and EVER-132-002 evaluated the efficacy and safety of SG versus treatment of physician’s choice (TPC) in patients with locally advanced inoperable HR+/HER2− mBC, after failure of at least two but no more than four prior chemotherapy regimens for metastatic disease.^14,15^ TROPiCS-02 primarily enrolled patients globally across 91 study centers in 9 countries including Belgium, Canada, France, Germany, Great Britain, Italy, Netherlands, Spain, and the United States, whereas EVER-132-002 was conducted across Asia (China mainland, Taiwan, and South Korea). The primary efficacy endpoint for both TROPiCS-02 and EVER-132-002 trials was progression-free survival (PFS), the secondary endpoints were overall survival (OS), objective response rate (ORR), and patient-reported outcomes, and additional endpoints included clinical benefit rate (CBR), duration of response (DOR), and safety.^14,15^ Additionally, in TROPiCS-02, patients were stratified based on visceral metastasis (yes/no), duration of endocrine therapy (⩾6 months vs not), and number of prior lines of therapy (two vs three or four), whereas, in EVER-132-002, patients were stratified based upon visceral metastasis, number of prior lines of therapy as well as prior cyclin-dependent kinase 4/6 inhibitor (CDK4/6i) treatment.^14,15^ We report a post hoc analysis of the two trials to compare the efficacy outcomes for SG versus TPC based on the pooled data of both the trial populations.

## Methodology

The meta-analysis was conducted in accordance with the Preferred Reporting Items for Systematic Reviews and Meta-Analyses of Individual Participant Data (PRISMA-IPD) guidelines.^16^

### Selection criteria

The randomized controlled phase III trials assessing SG versus TPC in patients with HR+/HER2− mBC who had previously received endocrine therapy, taxane, and at least two systemic therapies in the advanced setting were included in this study. Detailed information about the TROPiCS-02 and EVER-132-002 trials included in this study has been previously published.^13–15^ The eligibility criteria for SG versus TPC meta-analysis are summarized in Table 1.

**Table 1.** Selection criteria for meta-analysis.

Criteria	TROPiCS-02	EVER-132-002
Population (P)	Subjects with HR+/HER2− mBC who have progressed after endocrine therapy, taxane, CDK4/6i, and at least 2 systemic therapies in the advanced setting	Subjects with HR+/HER2− mBC who have progressed after endocrine therapy, taxane, and at least 2 systemic therapies in the advanced setting
Intervention (I)	SG	SG
Comparator (C)	TPC, i.e., gemcitabine, eribulin, capecitabine, vinorelbine	TPC, i.e., gemcitabine, eribulin, capecitabine, vinorelbine
Outcome (O)	Efficacy outcomes • PFS • OS • DOR • ORR • CBR	Efficacy outcomes • PFS • OS • DOR • ORR • CBR
Prior CDK4/6i exposure	Yes, mandatory	Not mandatory
Study design (S)	Randomized controlled trials	Randomized controlled trials
Study centers	Global (Belgium, Canada, France, Germany, Great Britain, Italy, Netherlands, Spain, and the United States)	Asian (China mainland, Taiwan, and South Korea)

CBR, clinical benefit rate; CDK4/6i, cyclin-dependent kinase 4/6 inhibitor; DOR, duration of response; HER2−, human epidermal growth factor receptor 2 negative; HR+, hormone receptor-positive; mBC, metastatic breast cancer; ORR, objective response rate; OS, overall survival; PFS, progression-free survival; SG, sacituzumab govitecan; TPC, treatment of physician’s choice.

### Risk-of-bias assessment

According to the *Cochrane Handbook for Systematic Reviews of Interventions*, the eligible randomized controlled trials (RCTs) were assessed for bias using the Cochrane Collaboration Risk of Bias Tool (RoB 2.0).^17^

### Feasibility assessment

Meta-analysis is built on an assumption of transitivity. In order to hold true for the assumption of transitivity, both trials involving direct comparisons need to be sufficiently similar.^18^ Therefore, a feasibility assessment of the meta-analytic framework was conducted by evaluating the availability of evidence and heterogeneity of prognostic factors at trial and patient levels, as well as the approaches to meta-analyze the effect sizes of SG versus TPC from the TROPiCS-02 and EVER-132-002 trials. The feasibility process involved (1) identification of heterogeneous variables using regression methods and seeking clinician feedback; (2) assessment of statistical heterogeneity; and (3) meta-analytic model selection based on outcome type and heterogeneity assessment.^19,20^ In chi-square analysis, a *p*-value less than 0.05 indicates statistical significance, while a *p*-value greater than 0.05 indicates no statistical significance. When interpreting *I*^2^ values, 0%–25% suggests no heterogeneity, 25%–50% suggests low heterogeneity, 50%–75% indicates moderate heterogeneity, and 75%–100% signifies high heterogeneity.^21^

In accordance with PRISMA-IPD guidelines, the IPD from both trials were assessed for integrity, consistency, baseline imbalances, and missing values.^16^ The availability of IPD enabled the evaluation of various subgroups based on effect modifiers, allowing us to determine if certain individuals benefit more from the intervention than others.

### Statistical analysis

The meta-analysis was conducted through IPD using multiple methods including one-stage and two-stage meta-regression models. A one-stage IPD meta-analysis involves pooling and evaluating patient-level data from multiple clinical trials, whereas the two-stage IPD meta-analysis is typically employed when the individual-level data from multiple studies are initially analyzed separately in each study, and then the aggregated level results generated from each study are combined in a second stage to generate an overall estimate.^22,23^ In the meta-regression models, differences in patient geography between the two trials cannot be adjusted; however, prior studies have suggested that this difference has no significant impact on survival outcomes in breast cancer.^24,25^

In the current meta-analysis, the one-stage approach was the primary analysis method (base-case) used for all outcomes. Covariate adjustment was employed for OS, PFS, and DOR to mitigate potential confounding variable influence. The clinically relevant and statistically significant covariates identified during the feasibility assessment have been summarized in Supplemental Table 1 along with the model results for statistically significant covariates. The covariate adjustments were not applied to the binary outcomes, that is, ORR and CBR due to convergence problems in the model, which could involve several factors such as having too few data points (sparse data) and high correlation between variables (multicollinearity). Because such model specification did not yield stable estimates, only univariate regression was conducted to estimate ORR and CBR. The hazard ratio (HR) and 95% CI of OS, PFS, and DOR were estimated using a stratified Cox proportional hazards regression analysis, while the Mantel–Haenszel model was used to estimate the rate ratios (RR) and 95% CI for ORR and CBR. The sensitivity analysis was performed using a two-stage fixed-effect model and a one-stage model without adjusting for covariates to test the robustness of the base-case results. The results of the sensitivity analysis are summarized in Table 3.

## Results

### Description of the trials and risk-of-bias assessment

**Figure 1.**
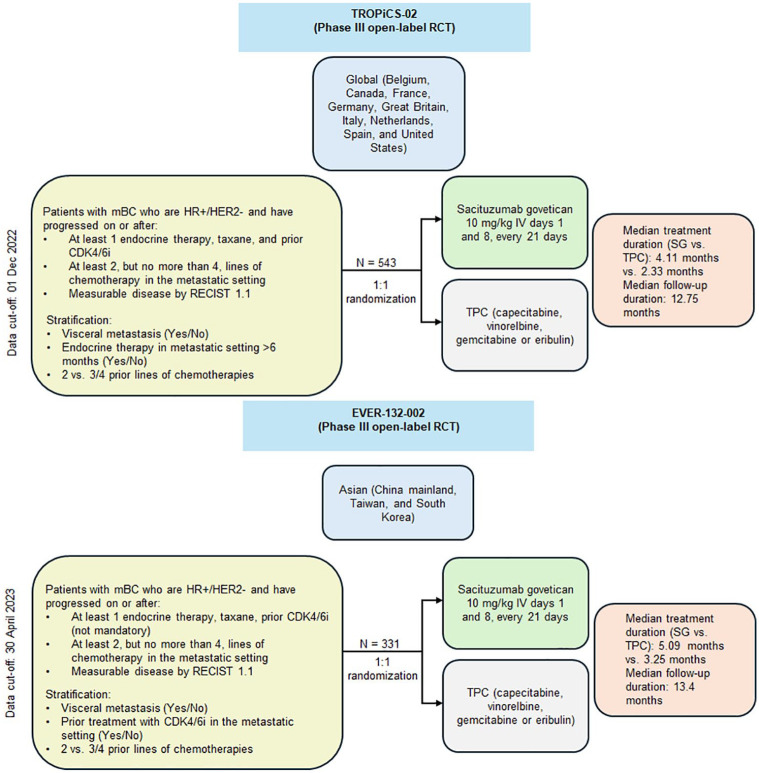
Trial characteristics. CDK4/6i, cyclin-dependent kinase 4/6 inhibitor; HER2−, human epidermal growth factor receptor 2 negative; HR+, hormone receptor-positive; mBC, metastatic breast cancer; RCT, randomized controlled trial; RECIST, Response Evaluation Criteria in Solid Tumors; SG, sacituzumab govitecan; TPC, treatment of physician’s choice.

Figure 1 presents the trial characteristics and Table 2 summarizes the baseline characteristics. A total of 1261 patients were recruited and 874 were randomized across both TROPiCS-02 and EVER-132-002. EVER-132-002 included 161 (48.6%) patients with prior CDK4/6i treatment status, whereas prior CDK4/6i treatment was a mandatory inclusion criterion for TROPiCS-02. The TROPiCS-02 trial included a substantially smaller proportion of Asian participants compared to EVER-132-002 (2.9% vs 100%). Regarding prior CDK4/6i treatment, the subgroup of prior CDK4/6i-treated subjects from EVER-132-002 was considered for feasibility and meta-analysis to match with the TROPiCS-02 for the primary analysis. Furthermore, a subgroup analysis was planned to explore the benefits of SG in the subgroup of patients who were treated with CDK4/6i for ⩽12 months (referred to as “fast-progressors”).

**Table 2.** Summary of baseline characteristics of studies.

Variable	TROPiCS-02 (*N* = 543)	EVER-132-002 (*N* = 331)
Overall population (irrespective of CDK4/6i treatment status)		
Age (%)	<65 years: 74.2 ⩾65 years: 25.8	<65 years: 88.5 ⩾65 years: 11.5
Mean age	56.4	52.1
Females (%)	99.1	99.4
Race (%)	American Indian or Alaska Native: 0 Asian: 2.9 Black or African American: 3.9 Multiple: 0.2 Native Hawaiian or Other Pacific Islander: 0.2 White: 66.7 Other: 0.6 Not reported: 25.6	American Indian or Alaska Native: 0 Asian: 100 Black or African American: 0 Multiple: 0 Native Hawaiian or Other Pacific Islander: 0 White: 0 Other: 0 Not reported: 0
Ethnicity (%)	Hispanic or Latino: 3.3 Not Hispanic or Latino: 78.5 Unknown: 5.2 Not reported: 11.2 Missing: 1.8	Hispanic or Latino: 0 Not Hispanic or Latino: 0 Unknown: 0 Not reported: 100 Missing: 0
Region (%)	North America: 42.2 Europe: 57.8 Asia: –	North America: – Europe: – Asia: 100
ECOG PS (%)	0: 44.4 1: 55.6	0: 22.4 1: 77.6
Baseline target/nontarget liver lesion per RECIST v1.1 (%)	Yes: 85.8 No: 14.2	Yes: 70.7 No: 29.3
Time from metastatic disease to randomization	Mean 54.4 months	Mean 43.6 months
Visceral disease (%)	Yes: 95.2 No: 4.8	Yes: 88.5 No: 11.5
Prior systemic anticancer regimens	Mean 7.4	Mean 6.0
Prior lines of chemotherapy in the metastatic setting (%)	>1: 98.0 1: 1.8	>1: 94.6 1: 5.4
Prior CDK4/6i use (%)	⩽12 months: 60.2 >12 months: 38.3 Missing: 1.5	⩽12 months: 32.9 >12 months: 15.7 Missing: 51.4
Pre-selected TPC choice (%)	Capecitabine: 8.1 Eribulin: 47.5 Vinorelbine: 21.4 Gemcitabine: 23.0	Capecitabine: 5.7 Eribulin: 81.9 Vinorelbine: 7.6 Gemcitabine: 4.8
Treatment distribution (%)	Capecitabine: 4.1 Eribulin: 23.9 Vinorelbine: 11.6 Gemcitabine: 10.3 SG: 50.1	Capecitabine: 3.3 Eribulin: 39.6 Vinorelbine: 3.9 Gemcitabine: 3 SG: 50.2
Number of prior systemic anticancer regimens by category (%)	1: 0.2 2: 13.6 3: 31.7 4: 33.3 5: 16.4 6: 4.1 7: 0.6 9: 0.2	1: – 2: 18.4 3: 39.6 4: 30.5 5: 10.9 6: 0.3 7: 0.3
Prior endocrine therapy in the metastatic setting for at least 6 months (%)	Yes: 86.4 No: 13.6	Yes: 77.6 No: 22.4
Early relapse (%)	Yes: 7.7 No: 89.9 Unknown: 2.4	Yes: 10.0 No: 89.4 Unknown: 0.6
Chemotherapy in neo/adjuvant setting (%)	Yes: 65.7 No: 34.3	Yes: 71.9 No: 28.1
Prior anthracycline use (%)	Yes: 79.7 No: 20.3	Yes: 83.7 No: 16.3
UGT1A1 genotype (in SG arm only) (%)	*1|*1: 38.2 *1|*6: – *27|*28: – *6|*28: – *6|*6: – *1|*28: 43.8 *1|*37: – *28|*28: 9.2 *28|*36: – Missing: 7.7	*1|*1: 46.6 *1|*6: 24.1 *27|*28: 1.2 *6|*28: 1.8 *6|*6: 3.0 *1|*28: 10.8 *1|*37: – *28|*28: 1.8 *28|*36: – Missing: 10.2

In TROPiCS-02, participants were from United States (42.0%), France (25.2%), Spain (12.7%), Germany (8.5%), Belgium (4.6%), Italy (2.8%), Great Britain (2.6%), Netherlands (1.5%), Canada (0.2%).

In EVER-132-002, participants were from the geographical regions of China (70.1%), South Korea (21.1%), and Taiwan (8.8%).

CDK4/6i, cyclin-dependent kinase 4/6 inhibitor; ECOG PS, Eastern Cooperative Oncology Group Performance status; HER2, human epidermal growth factor receptor 2; IHC, immunohistochemistry; RECIST, Response Evaluation Criteria in Solid Tumors; SG, sacituzumab govitecan; TPC, treatment of physician’s choice.

The mean age was comparable across both trials with a majority of the population having <65 years of age, an Eastern Cooperative Oncology Group Performance status (ECOG PS) of 1, and having received more than one prior line of chemotherapy in the metastatic setting (Table 2). The differences observed between both studies in the patient characteristics were confirmed further using outcome-specific univariate and multivariate regression analysis. The statistical heterogeneity assessment for survival and response rate outcomes indicated low to moderate heterogeneity between TROPiCS-02 and EVER-132-002 trials among the overall and prior CDK4/6i-treated patients (ranging from 0% to 70.0%), which allowed proceeding toward the statistical analysis stage; however, the heterogeneity was higher for CBR (76.9%) in the prior CDK4/6i-treated patients.

Both studies were open-label, phase III RCTs with an overall low risk of bias across multiple domains including randomization process, deviations from intended interventions, missing outcome data, measurement of the outcome, and selection of the reported result.

### Progression-free survival

**Figure 2.**
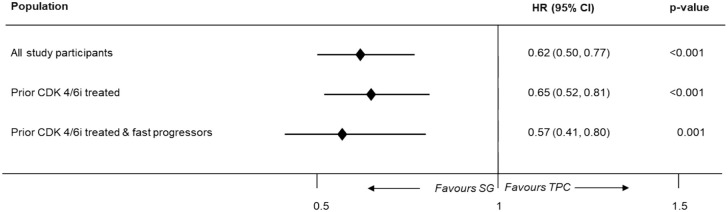
Hazard ratios for PFS of patients with HR+/HER2− mBC using the one-stage approach; pooled data from two trials. CDK4/6i, cyclin-dependent kinase 4/6 inhibitor; CI, confidence interval; HER2−, human epidermal growth factor receptor 2 negative; HR+, hormone receptor-positive; HR, hazard ratio; mBC, metastatic breast cancer; PFS, progression-free survival; SG, sacituzumab govitecan; TPC, treatment of physician’s choice.

A statistically significantly improved PFS was observed with SG compared to TPC using the one-stage method across the overall population (median PFS, 5.30 months (95% CI: 4.20–5.70) vs 4.10 months (95% CI: 2.90–4.20); log-rank *p*-value = 0.0001), prior CDK4/6i-treated population (median PFS, 4.70 months (95% CI: 4.10–5.70) vs 3.70 months (95% CI: 2.80–4.20); log-rank *p*-value = 0.0005) and fast-progressors (median PFS, 5.60 months (95% CI: 4.20–6.90) vs 3.20 months (95% CI: 2.80–4.20); log-rank *p*-value = 0.004). Figure 2 depicts the HR values for PFS using the one-stage approach.

### Overall survival

**Figure 3.**
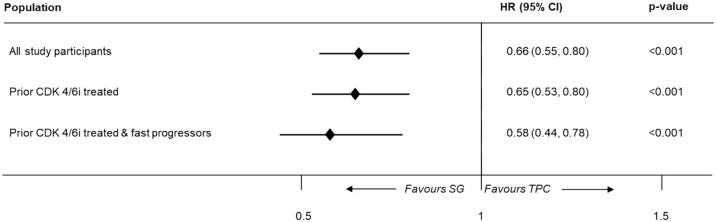
Hazard ratios for OS of patients with HR+/HER2− mBC using the one-stage approach; pooled data from two trials. CDK4/6i, cyclin-dependent kinase 4/6 inhibitor; CI, confidence interval; HER2−, human epidermal growth factor receptor 2 negative; HR+, hormone receptor-positive; HR, hazard ratio; mBC, metastatic breast cancer; OS, overall survival; SG, sacituzumab govitecan; TPC, treatment of physician’s choice.

In the overall population, irrespective of prior CDK4/6i status, a statistically significantly better OS was observed with SG compared to TPC using the base-case, one-stage method (median OS, 16.20 months (95% CI: 14.85–18.07) vs 12.71 months (95% CI: 11.50–13.86); log-rank *p*-value < 0.001). Similar findings were reported in the subgroup of patients previously treated with CDK4/6i (median OS, 15.38 months (95% CI: 14.03–16.92) vs 11.50 months (95% CI: 10.64–12.87); log-rank *p*-value < 0.001) and in fast-progressors (median OS, 16.0 months (95% CI: 14.32–18.23) vs 11.10 months (95% CI: 10.09–12.48); log-rank *p*-value = 0.001). Figure 3 depicts the HR values for OS using the one-stage approach.

### Response outcomes

**Figure 4.**
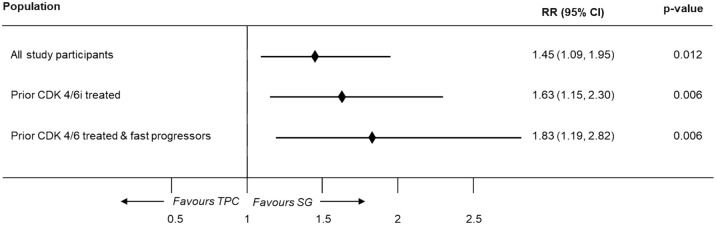
Meta-analysis results for ORR (one-stage). CDK4/6i, cyclin-dependent kinases 4 and 6 inhibitors; CI, confidence interval; ORR, objective response rate; RR, rate ratio; SG, sacituzumab govitecan; TPC, treatment of physician’s choice.

**Figure 5.**
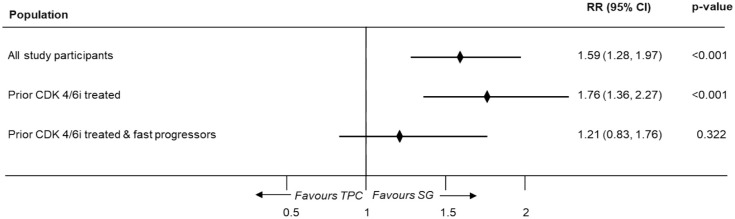
Meta-analysis results for CBR (one-stage). CBR, clinical benefit rate; CDK4/6i, cyclin-dependent kinases 4 and 6 inhibitors; CI, confidence interval; RR, rate ratio; SG, sacituzumab govitecan; TPC, treatment of physician’s choice.

Compared to TPC, SG exhibited a statistically significantly higher ORR in the overall (RR, 1.45 (95% CI: 1.09–1.95); *p* = 0.012), prior CDK4/6i-treated (RR, 1.63 (95% CI: 1.15–2.30); *p* = 0.006), and fast-progressors population (RR, 1.83 (95% CI: 1.19–2.82); *p* = 0.006) using the one-stage approach (Figure 4). A similar trend was observed in pooled results of CBR, where SG showed a higher CBR compared to TPC in overall, prior CDK4/6i treated & fast progressors population (Figure 5).

### Duration of response

**Figure 6.**
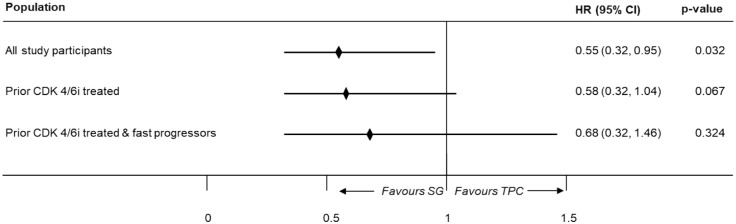
Meta-analysis results for DOR (one-stage). CDK4/6i, cyclin-dependent kinases 4 and 6 inhibitors; CI, confidence interval; DOR, duration of response; HR, hazard ratio; SG, sacituzumab govitecan; TPC, treatment of physician’s choice.

In the overall population, SG showed a significantly longer DOR compared to TPC (HR, 0.55 (95% CI: 0.32–0.95); *p* = 0.032) using the one-stage method. Furthermore, SG showed a longer, but marginally non-significant DOR compared to TPC among prior CDK4/6i-treated (HR, 0.58 (95% CI: 0.32–1.04); *p* = 0.067) and fast-progressors population (HR, 0.68 (95% CI: 0.32–1.46); *p* = 0.324) due to lower number of patients with complete response or partial response in both trials (*n* = 155, 116 and 76 for overall, prior CDK4/6i-treated, and fast-progressors population, respectively; Figure 6).

### Sensitivity analysis

The sensitivity analyses were performed employing a two-stage fixed-effect model for all the outcomes and a one-stage model without covariates for OS, PFS, and DOR to test the robustness of the base-case results. The covariate adjustments were not applied to ORR and CBR due to convergence problems in the model, which could involve factors such as having too few data points or sparse data and high correlation between variables. These results were broadly aligned with the base-case findings, demonstrating that SG was associated with statistically significantly better efficacy compared to TPC (Table 3).

**Table 3.** Summary of the results for one-stage (base-case), two-stage, and one-stage without covariates for all three populations.

Type of analysis	Overall population	Prior CDK4/6i treated	Prior CDK4/6i treated and fast-progressors
	**HR (95% CI), ***p***-value**		
Progression-free survival; HR (95% CI), *p*-value			
One-stage with covariates (base-case)	0.62 (0.50, 0.77), <0.001	0.65 (0.52, 0.81), <0.001	0.57 (0.41, 0.80), 0.001
Fixed-effect model (two-stage)	0.67 (0.55, 0.83), <0.001	0.66 (0.52, 0.84), 0.001	0.58 (0.43, 0.73), <0.001
One-stage without covariates	0.68 (0.57, 0.80), <0.001	0.64 (0.53, 0.77), <0.001	0.58 (0.46, 0.74), <0.001
Overall survival; HR (95% CI), *p*-value			
One-stage with covariates (base-case)	0.66 (0.55, 0.80), <0.001	0.65 (0.53, 0.80), <0.001	0.58 (0.44, 0.78), <0.001
Fixed-effect model (two-stage)	0.70 (0.58, 0.86), <0.001	0.68 (0.55, 0.84), <0.001	0.68 (0.44, 0.91), <0.001
One-stage without covariates	0.74 (0.63, 0.87), <0.001	0.73 (0.62, 0.87), <0.001	0.62 (0.50, 0.77), <0.001
Duration of response; HR (95% CI), *p*-value			
One-stage with covariates (base-case)	0.55 (0.32, 0.95), 0.032	0.58 (0.32, 1.04), 0.067	0.68 (0.32, 1.46), 0.324
Fixed-effect model (two-stage)	0.48 (0.27, 0.84), 0.011	0.53 (0.27, 1.01), 0.055	0.75 (0.11, 1.39), 0.021
One-stage without covariates	0.67 (0.42, 1.06), 0.089	0.67 (0.39, 1.15), 0.144	0.50 (0.19, 1.34), 0.169
Objective response rate; RR (95% CI), *p*-value			
One-stage^a^ (base-case)	1.45 (1.09, 1.95), 0.012	1.63 (1.15, 2.30), 0.006	1.83 (1.19, 2.82), 0.006
Fixed-effect model (two-stage)	1.45 (1.09, 1.95), 0.012	1.63 (1.15, 2.30), 0.006	1.82 (0.99, 2.65), 0.000
Clinical benefit rate; RR (95% CI), *p*-value			
One-stage^a^ (base-case)	1.59 (1.28, 1.97), <0.001	1.76 (1.36, 2.27), <0.001	1.21 (0.83, 1.76), 0.322
Fixed-effect model (two-stage)	1.59 (1.28, 1.97), <0.001	1.76 (1.36, 2.27), <0.001	1.23 (0.85, 1.77), 0.277

a The covariate adjustments were not applied to ORR and CBR due to convergence problems in the model.

CBR, clinical benefit rate; CDK4/6i, cyclin-dependent kinase 4/6 inhibitors; CI, confidence interval; HR, hazard ratio; ORR, objective response rate; RR, rate ratio.

## Discussion

The current study was conducted to meta-analyze efficacy outcomes between SG and TPC in patients with HR+/HER2− mBC using TROPiCS-02 and EVER-132-002 trials. The multicenter and multiregional population evaluated in the study indicates the possibility of global generalizability, with proven clinical benefits of SG confirming similar assertions made in a recently published review paper.^26^ Our analysis was conducted in accordance with published guidelines^16^ and based on IPD methodology. While baseline parameters across both trials were generally similar, variability was reported in the region and prior CDK4/6i use. The findings of the meta-analysis demonstrated that SG significantly improved PFS, OS, and response outcomes compared to TPC. These benefits were observed consistently across various subgroups, including patients pre-treated with CDK4/6i and fast-progressors.

In the base-case one-stage analysis, SG showed statistically significantly better OS as compared to TPC in the overall, prior CDK4/6i-treated, and fast-progressors populations. Sensitivity analyses using the two-stage fixed-effect model and a one-stage without covariates model were completely aligned with the base-case one-stage results. Like OS, SG showed statistically significantly better PFS compared to TPC in the overall population as well as prior CDK4/6i-treated and fast-progressors subgroups. Furthermore, SG showed better response duration as compared to TPC, in the overall population. Although DOR results among the prior CDK4/6i-treated and fast-progressor subgroups were directionally aligned with the overall results, statistical significance was not achieved. It should be noted that the DOR was measured only among the subset of patients who achieved an objective response, resulting in a smaller sample size that might not be adequately powered to detect statistically significant differences. Consequently, DOR can primarily be used to indicate the direction of benefit, which, in our study, favors SG compared to TPC among all analysis sets. The results of the IPD-based meta-analysis revealed a statistically significantly higher ORR and CBR with SG compared to TPC in overall and prior CDK4/6i-treated patients; however, the CBR results for fast-progressors were numerically favoring SG. The two-stage and one-stage results of efficacy outcomes were consistent except for DOR in the fast-progressors subgroup where results significantly favored SG in two-stage but were non-significant in one-stage.

The findings of our analysis were similar to a recent systematic review and meta-analysis conducted by Qureshi et al.,^27^ where treatment with SG resulted in a significant reduction in the risk of disease progression and improved survival. Moreover, the findings aligned with individual trial results of TROPiCS-02 and EVER-132-002, where both showed improved efficacy in HR+/HER2− mBC. In the TROPiCS-02 trial, SG demonstrated statistically significant and clinically meaningful benefits in OS among patients with pre-treated, endocrine-resistant HR+/HER2− mBC.^13,28,29^ Similar findings were reported in the EVER-132-002 trial.^15^ A statistically significant PFS benefit with SG over single-agent chemotherapy was observed in both these trials. Details of findings from the individual trial have been provided in Supplemental Table 2. Furthermore, similar results of sensitivity analyses confirmed the robustness of the base-case findings, with consistent results observed across different statistical models. This underscores the reliability of the primary analysis and strengthens the evidence supporting SG’s clinical benefits.

Our results could be contextualized considering efficacy findings associated with other ADCs including trastuzumab deruxtecan (T-DXd) and datopotamab deruxtecan (Dato-DXd) evaluated in DESTINY-Breast04 and TROPION-Breast01, respectively. DESTINY-Breast04 is a phase III trial involving patients with HER2-low, unresectable, or mBC who had received chemotherapy for metastatic disease or have had disease recurrence during or within 6 months after completing adjuvant chemotherapy, while the patients with HR+ disease must have received at least one line of endocrine therapy.^30^ On the other hand, TROPION-Breast01 is a phase III trial involving patients with inoperable or metastatic HR+/HER2− breast cancer who have received one or two prior lines of systemic chemotherapy.^31^ In the DESTINY-Breast04 trial, T-DXd successfully prolonged both PFS (HR, 0.50 (95% CI: 0.40–0.63); *p* < 0.001) and OS (HR, 0.64 (95% CI: 0.49–0.84); *p* = 0.001) among patients with HER2-low mBC compared to physician’s choice of chemotherapy. Moreover, the proportion of patients with a confirmed objective response among all patients was also higher in the T-DXd group versus the physician’s choice group (52.3% (95% CI: 47.1–57.4) vs 16.3% (95% CI: 11.3–22.5)). Similar findings were reported for the subgroup of patients with HR-positive disease (PFS: HR, 0.51 (95% CI: 0.40–0.64); *p* < 0.001, OS: HR, 0.64 (95% CI: 0.48–0.86); *p* = 0.003, ORR: 52.6% (95% CI: 47.0–58.0) vs 16.3% (95% CI: 11.0–22.8) respectively).^30^ The findings from the TROPION-Breast01 trial reported that patients receiving Dato-DXd had significantly improved PFS versus investigator’s choice of chemotherapy (HR, 0.63 (95% CI: 0.52–0.76); *p* < 0.0001). For the OS, interim results numerically favored Dato-DXd versus chemotherapy (HR, 0.84 (95% CI: 0.62–1.14)); however, statistical significance was not reached since the OS data were not mature. The results also demonstrated a confirmed ORR of 36.4% in patients treated with Dato-DXd compared to an ORR of 22.9% with chemotherapy.^31,32^ The current meta-analysis yielded pooled treatment effects (i.e., HR_PFS_ and HR_OS_) comparing SG versus TPC which were found to be within 95% CI of the respective measures obtained in DESTINY-Breast04 and TROPION-Breast-01. The meta-analysis results favor SG versus single-agent chemotherapy, particularly in later lines of treatment, thereby establishing it as an effective treatment strategy in patients with limited treatment options. However, since differences in baseline patient characteristics were not adjusted among the different trials, cross-trial comparison of treatment effects should be done with caution.

The strengths of the meta-analysis include the rigorous adherence to PRISMA-IPD guidelines, ensuring a systematic and transparent approach to data synthesis. The use of IPD allowed for detailed subgroup analyses and adjustment for relevant covariates, enhancing the robustness of the findings. Additionally, the feasibility assessment confirmed the validity of pooling data from the TROPiCS-02 and EVER-132-002 trials, which were sufficiently similar in design and patient characteristics. However, this study also had a few limitations. For example, the variability in patient geography between the trials, particularly the higher proportion of Asian patients in the EVER-132-002 trial, could introduce unmeasured confounding, although there is a lack of documented evidence to substantiate such bias. Covariate adjustments for binary outcomes (ORR and CBR) could not be performed due to convergence problems, likely caused by sparse data and multicollinearity. Potential issues with missing data, baseline imbalances, and inconsistencies in the IPD may have affected the reliability of the results. Moreover, differences between one-stage and two-stage meta-analysis approaches could impact results, especially for covariate adjustments. The Cox model assumes proportional hazards, which, if violated, could bias estimates for survival outcomes. The inclusion of only two trials might introduce publication bias if other trials with differing results were not considered. Lastly, the analysis focused on patients with HR+/HER2− mBC, which limits the generalizability of the findings to other breast cancer subtypes (e.g., mTNBC or HER2+). While SG showed improvements in efficacy outcomes for this specific population, the findings may not apply to broader or more heterogeneous breast cancer populations.

To our knowledge, this research is the first meta-analytic study evaluating the clinical benefits of an ADC in the HR+/HER2− mBC population.^33^ ADCs represent a class of targeted anticancer therapies that have garnered significant attention and development in recent years. They combine the specificity of monoclonal antibodies with the potency of cytotoxic drugs, designed to minimize harm to healthy tissues by delivering chemotherapy with enhanced specificity.^34^ The suite of analyses completed in our study is similar to what has been completed by previous researchers while evaluating the efficacy of another HER2-targeting ADC (trastuzumab emtansine) in improving outcomes of HER2-positive locally advanced or mBC patients.^35^ Such similarity in methodological approach across different studies confirms the usefulness of meta-analytic frameworks in increasing evidence base while performing comparative effectiveness research. Even though safety analysis was outside the scope of our study, additional research for evaluating the benefit versus risk of choice of SG as treatment selection from a patient perspective may be warranted.

## Conclusion

In conclusion, this meta-analysis confirms that SG significantly improves clinical outcomes in patients with HR+/HER2− mBC, including those pre-treated with CDK4/6i and fast-progressors when compared to TPC. These findings extend previous research, supporting the integration of SG into clinical practice guidelines at a global level for treating HR+/HER2− mBC population irrespective of status and duration of prior CDK4/6i exposure.

## Supplemental Material

sj-docx-1-tam-10.1177_17588359251320285 – Supplemental material for Efficacy of sacituzumab govitecan versus treatment of physician’s choice in previously treated HR+ and HER2− mBC: a meta-analysis of TROPiCS-02 and EVER-132-002 trials Supplemental material, sj-docx-1-tam-10.1177_17588359251320285 for Efficacy of sacituzumab govitecan versus treatment of physician’s choice in previously treated HR+ and HER2− mBC: a meta-analysis of TROPiCS-02 and EVER-132-002 trials by Oleg Gluz, Binghe Xu, Rita Nanda, Anandaroop Dasgupta, Ankita Kaushik, Wendy Verret, Akanksha Sharma, Barinder Singh and Hope S. Rugo in Therapeutic Advances in Medical Oncology

sj-docx-2-tam-10.1177_17588359251320285 – Supplemental material for Efficacy of sacituzumab govitecan versus treatment of physician’s choice in previously treated HR+ and HER2− mBC: a meta-analysis of TROPiCS-02 and EVER-132-002 trials Supplemental material, sj-docx-2-tam-10.1177_17588359251320285 for Efficacy of sacituzumab govitecan versus treatment of physician’s choice in previously treated HR+ and HER2− mBC: a meta-analysis of TROPiCS-02 and EVER-132-002 trials by Oleg Gluz, Binghe Xu, Rita Nanda, Anandaroop Dasgupta, Ankita Kaushik, Wendy Verret, Akanksha Sharma, Barinder Singh and Hope S. Rugo in Therapeutic Advances in Medical Oncology

## Declarations

**Ethics approval and consent to participate:** Not applicable. The study was a secondary analysis of published papers and did not involve additional human subjects.

**Consent for publication:** Not applicable.

**Availability of data and materials:** Additional data regarding the results were included in the Supplemental Material. Additional data not included in the Supplemental Material are available on request from the corresponding author.
