# Supplementary material for: Efficacy of sacituzumab govitecan versus treatment of physician’s choice in previously treated HR+ and HER2− mBC: a meta-analysis of TROPiCS-02 and EVER-132-002 trials
Source: Ther Adv Med Oncol. 2025 Mar 14;17:17588359251320285. doi: 10.1177/17588359251320285 (PMC11907608; doi:10.1177/17588359251320285)
Supplement: sj-docx-1-tam-10.1177_17588359251320285 – Supplemental material for Efficacy of sacituzumab govitecan versus treatment of physician’s choice in previously treated HR+ and HER2− mBC: a meta-analysis of TROPiCS-02 and EVER-132-002 trials [file sj-docx-1-tam-10.1177_17588359251320285.docx]

# Supplementary Materials

Table 1: List of clinically and statistically significant covariates identified during feasibility assessment for the overall population (one-stage)

| **Variables (Values vs. Reference)** | **HR (95% CI)** | **P-value** |
| --- | --- | --- |
| **Overall survival** | | |
| Race (Asian vs. White) | 0.57 (0.46, 0.70) | 0 |
| Screening ECOG Performance Status (1: Symptoms but Ambulatory vs. 0: Normal Activity) | 1.36 (1.14, 1.61) | <0.001 |
| Time from Metastatic breast cancer to Randomization (>50 vs. <50 months) | 0.66 (0.55, 0.79) | 0 |
| Liver Metastasis at Baseline (Yes vs. No) | 1.62 (1.28, 2.05) | <0.001 |
| Treatment Physician Choice (Vinorelbine vs. Capecitabine) | 1.66 (1.17, 2.37) | 0.005 |
| Endo-Therapy in Metastatic Setting (Yes vs No) | 0.75 (0.60, 0.94) | 0.012 |
| Geographic Region (Europe vs. China) | 1.47 (1.18, 1.84) | <0.001 |
| Geographic Region (North America vs. China) | 1.47 (1.16, 1.87) | 0.002 |
| Visceral Disease (Yes vs. No) | 1.69 (1.16, 2.45) | 0.006 |
| **Progression-free survival** | | |
| Age (50-64 vs. 27-49 years) | 0.80 (0.66, 0.96) | 0.017 |
| Race (Asian vs. White) | 2.11 (1.50, 2.98) | 0 |
| Time from Metastatic breast cancer to Randomization (>50 vs. <50 months) | 0.77 (0.65, 0.93) | 0.005 |
| Liver Metastasis at Baseline (Yes vs. No) | 1.38 (1.08, 1.77) | 0.011 |
| Prior CDK4/6i use (No vs. Yes) | 0.73 (0.57, 0.95) | 0.018 |
| Number of Prior Chemo Regimen (4 or 5 vs. 2 or 3) | 1.21 (1.02, 1.43) | 0.027 |
| Endo-Therapy in Metastatic Setting (Yes vs. No) | 0.77 (0.62, 0.97) | 0.023 |
| Age (65-86 vs. 27-49 years) | 0.75 (0.59, 0.94) | 0.015 |
| **Duration of response** | | |
| Race (Asian vs. White) | 17.04 (1.47, 197.35) | 0.023 |
| Time from Metastatic breast cancer to Randomization (>50 vs. <50 months) | 0.52 (0.31, 0.89) | 0.016 |
| Geographic Region (South Korea vs. China) | 0.35 (0.12, 0.99) | 0.048 |
| CI: Confidence interval; HR: Hazards ratio | | |

Table 2: Overall survival and progression-free survival results for patients with HR+/HER2- mBC for individual trials

| **Population** | **TROPiCS-02**  **HR (95% CI)** | **EVER-132-002**  **HR (95% CI)** |
| --- | --- | --- |
| **Overall Survival** | | |
| Overall population | 0.79 (0.65, 0.96) | 0.64 (0.47, 0.88) |
| Prior CDK4/6i-treated | 0.79 (0.65, 0.96)* | 0.50 (0.31, 0.80) |
| Prior CDK4/6i-treated and fast-progressors | 0.68 (0.53, 0.88) | 0.44 (0.25, 0.75) |
| **Progression-free survival** | | |
| Overall population | 0.66 (0.53, 0.83) | 0.67 (0.52, 0.87) |
| Prior CDK4/6i-treated | 0.66 (0.53, 0.83)* | 0.56 (0.39, 0.81) |
| Prior CDK4/6i-treated and fast-progressors | 0.59 (0.44, 0.78) | 0.52 (0.34, 0.81) |
| CDK4/6i: CDK4/6 inhibitors; CI: Confidence interval; HR: Hazard ratio; mBC: Metastatic breast cancer; NA: Not applicable  *prior CDK4/6i use was a mandatory inclusion criterion in TROPiCS | | |
